# The Importance of Branch Placement on the Dilute Solution Properties of Comb-like Macromolecules

**DOI:** 10.1021/acs.macromol.5c00323

**Published:** 2025-06-12

**Authors:** Robert J. S. Ivancic, Chase B. Thompson, Devin A. Golla, Bintou Koroma, Jack F. Douglas, Sara V. Orski, Debra J. Audus

**Affiliations:** † Materials Science and Engineering Division, 10833 National Institute of Standards and Technology, Gaithersburg, Maryland 20899, United States; § Department of Chemical and Biomolecular Engineering, 6572 University of Pennsylvania, Philadelphia, Pennsylvania 19104, United States; ∥ Fischell Department of Bioengineering, 1068 University of Maryland, College Park, Maryland 20742, United States

## Abstract

Branch density and length substantially impact the properties of comb-like polymers. Scientists often use the dilute solution properties of these materials to quantify their architecture. As branch spacing decreases and branch length increases at a fixed molecular mass, dilute solution properties such as the radius of gyration, intrinsic viscosity, and hydrodynamic radius typically decrease because the length of the backbone decreases. However, this decrease is only partially driven by this change in backbone length, even for relatively short branches. While many models focus on predicting the dilute solution properties of these materials with fixed branch spacing, most comb-like polymers exhibit statistical branch spacing which leads to nontrivial changes in excluded volume effects. Using molecular dynamics simulations and the ZENO code, we show how changing the distribution of branches from fixed to statistical and then to diblock affects the dilute solution properties of a coarse-grained linear low-density polyethylene (LLDPE), a canonical comb-like polymer, in 1,2,4-trichlorobenzene, a standard good solvent. This approach explicitly accounts for excluded volume interactions that were not included in prior theories. We extend our previous theoretical work to account for statistical branch spacing and test prior renormalization group estimates of diblocks in good solvent to show that it is consistent with our numerical results. Our approach provides a framework for a more quantitative understanding of chain architecture from dilute solution properties, yielding better structure–property relationships.

## Graphical abstract

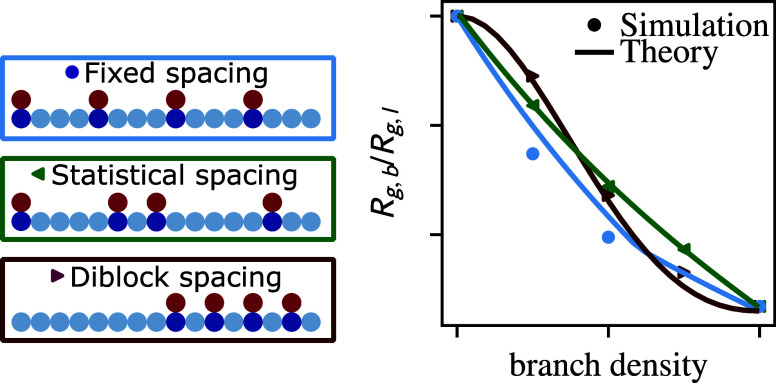

## Introduction

Comb-like polymers, linear polymers with linear side chains, have various applications, such as conductive textiles,^1^ paint,^2^ drug delivery,^3^ and chemotherapy.^4^ One reason these polymers are attractive is that varying the length and spacing of the side chains can lead to substantial changes in properties.^5,6^ A classic, industrially important example of this effect is linear low-density polyethylene (LLDPE). As the side chains get longer, LLDPE impact strength, ductility, and impact fatigue life increase.^7,8^ As the density of side chains increases, i.e., the average branch spacing decreases, melt temperature, crystallization temperature, and lamella thickness decrease precipitously.^9^ While the architecture of comb-like polymers is critical to their resulting properties, accessing their detailed structure can be challenging even with well-known synthesis techniques. Thus, the development of better methods to probe this structure is essential to making better materials and characterizing them accurately.

One way to interrogate the structure of polymers is to take a sample, dissolve it in a good solvent, and then investigate its dilute solution properties, such as its radius of gyration (*R*_g_), intrinsic viscosity ([η]), and hydrodynamic radius (*R*_h_). In a good solvent, these properties scale as

1 $$p=K_{p}M^{\nu_{p}}$$

where *M* is the molar mass, ν_p_ is the scaling exponent of property *p* (= *R*_g_, [η], or *R*_h_) and *K*_p_ is a prefactor. In the case of intrinsic viscosity, this function is known as the Kuhn–Mark–Houwink–Sakurada equation.^10^ We expect linear polymers with large hydrodynamic interactions to scale as ν_*R*_g__ ≈ 0.588, ν_[η]_ ≈ 0.7, and ν_*R*_h__ ≈ 0.6 in a good solvent.^11–13^

Chain architecture can dramatically affect these properties. A standard method to quantify these changes is examining the contraction factor of property p (*g*_p_), the ratio of branched to linear dilute solution properties at the same mass. This representation enhances property changes resulting from the chain architecture, in this case branching. Specifically, we consider

2 $$g_{p}=\frac{p_{b}}{p_{l}}$$

where the subscripts *l* and *b* denote whether we measure the property for the corresponding/equivalent linear or branched polymers, respectively. This notation follows that of Douglas and co-workers. We point out that most theoretical work on these quantities involves the assumption of Gaussian chains and a hydrodynamic preaveraging approximation, making them somewhat unreliable.^14,15^ Moreover, the mass dependence of this equation drops out if the scaling exponents (ν_p_) are the same for the branched and linear polymers. While it is widely observed that these factors are less than unity for many comb-like polymers due to the increased linear density of the chain, the exact origin of this decrease remains poorly understood.^16–18^ This lack of understanding hinders the mapping of dilute solution properties to their underlying comb-like architecture, thereby limiting the use of this method for probing chain architecture.

Theorists have previously proposed several useful scaling models to address this challenge. These models often attempt to predict the polymer’s radius of gyration based on the Flory approximation, which assumes strong hydrodynamic interactions and a linear scaling between different measures of polymer size. They imply scaling relationships in powers of the branch length (*L*) and spacing (*S*), i.e., *L* ^β_*L*_^ *S* ^β_*S*_^.^19–22^ These theories often distinguish between the comb-like regime in which branches weakly interact, which is of interest here, and the bottlebrush regime (*L* ≫ *S*) in which branches strongly interact. Recently, Pan and co-workers tested many of these models experimentally, finding that *R*_g_ ∼(*L*/*S*)^0.37^.^23,24^ Molecular dynamics simulations^20,25^ have also proven valuable tools to test these theories.

In addition to the above theories, we previously introduced a semiempirical model for comb-like polymers with precise branch spacings (*S*) and lengths (*L*). We subsequently tested our model against molecular dynamics simulations.^26^ This model accounts for changes in the persistence length from two effects. As with previous models, it suggests a scaling of the radius of gyration with increasing branch length but also incorporates short-ranged repulsion between the backbone and the branch directly into the model. Moreover, it uses empirical scaling relationships to predict intrinsic viscosity and the hydrodynamic radius from the radius of gyration.

While these models offer predictions for the dilute solution properties of polymers, they assume fixed branch spacing or do not depend on the placement of branches along the backbone. Although carefully designed experiments meet the condition of fixed branch spacing,^17^ most industrial comb-like polymers, e.g., LLDPE, and many experimental architectures^22,23,27^ have side chains placed in an uncontrolled or statistical manner along the backbone. To investigate whether branch placement changes dilute solution properties, Thompson and Orski synthesized two sets of LLDPE, diblocks and statistical copolymers of LLDPE to investigate this effect further.^28^ They synthesized the diblocks using a one-pot sequential ring-opening metathesis polymerization of 1-butyl-*trans*-cyclooctene and 1,5-*cis*,*cis*-cyclooctadiene. This chemistry generates polymers that have a branch spacing of exactly eight carbons and a branch length of four carbons for one of the blocks, while the other block is linear polyethylene. The statistical copolymers also had a branch length of four carbons and a minimum branch spacing of eight carbons. When they compared these two polymer architectures at the same branch density, they exhibited substantially different intrinsic viscosities.

On a theoretical level, it is unsurprising that branch placement has a substantial effect on the dilute solution properties of polymers. Though few have considered the dilute solution properties of statistical copolymers, many have looked into the more tractable problem of diblocks. Mondescu and Muthukumar have developed an analytical theory for many dynamical properties of diblock copolymers in good solvents.^29^ Douglas and Freed have considered a renormalization group description for the radius of gyration in a good solvent,^30^ and their solution was consistent with earlier Monte Carlo simulation estimates by Tanaka et al.^31^ These results and recent experiments^28,32^ suggest that the distribution of branches along the polymer backbone plays a role in determining the dilute solution properties of a given polymer architecture.

Here, we summarize our model for predicting dilute solution properties of comb-like macromolecules with fixed branch spacing and length.^26^ We build upon this work to design a model for statistically placed branches similar to many industrial cases. Although this model does not incorporate branch spacing gradients or correlations between branches, it serves as an approximation of these more realistic materials. We then outline how to apply the work of Douglas and Freed^30^ to predict the radius of gyration of diblock polymers in the context of diblock linear-comb polymers. We test these theories by comparing them against a coarse-grained, implicit solvent model of LLDPE in 1,2,4-trichlorobenzene, a canonical polymer-good solvent pair developed in our previous work.^26^ The LLDPE we consider is in the comb-like rather than bottlebrush regime (*L* ≫ *S*). We first show that this potential semiquantitatively reproduces our intrinsic viscosity experiments for diblock and statistical LLDPE.^28^ We then show that the described theories are consistent with the data. Finally, we test the relationship between the freely jointed chain persistence length and the contraction factors, relating chain-level properties directly to the microscopic, freely jointed chain model persistence length.

## Methods

We investigate the dilute solution properties of LLDPE in good solvent with different branch spacing distributions using a previously developed coarse-grained molecular dynamics model.^26^ This model was designed to replicate all-atom bond length, angle, and dihedral distributions of LLDPE in 1,2,4-trichlorobenzene, a canonical good solvent, at a temperature of *T* = 135 °C and pressure of *P* = 101 kPa (1 atm). Thus, it captures the small-length scale properties of the polymer neglected in previous modeling of these polymers. Moreover, the scaling of its intrinsic viscosity and radius of gyration extrapolate to experimental LLDPE scaling relationships of LLDPE with a branch spacing of *S* = 8 carbons and branch lengths of *L* = 0 (linear polyethylene), 2, 4, 6, and 10 carbons as studied by Orski and co-workers.^17^ This observation indicates the model captures the good solvent properties of LLDPE at large length scales. This model has three coarse-grained beads, as shown in Figure 1A. Main chain monomers (light blue, A) are ethylenes with four carbons. Branch monomers (dark blue, B) are the same as main chain monomers, but they contain a methanetriyl group, i.e., an extra carbon bond, on the third carbon atom to allow for branching. Connector monomers attach to branch monomers using these extra carbon bonds. These monomers are ethylenes with four carbons (dark red, *C*_4_). The parameters for these units’ bond, angle, dihedral, and nonbonded potentials are provided in the Supporting Information of our prior manuscript.^26^

**Figure 1:**
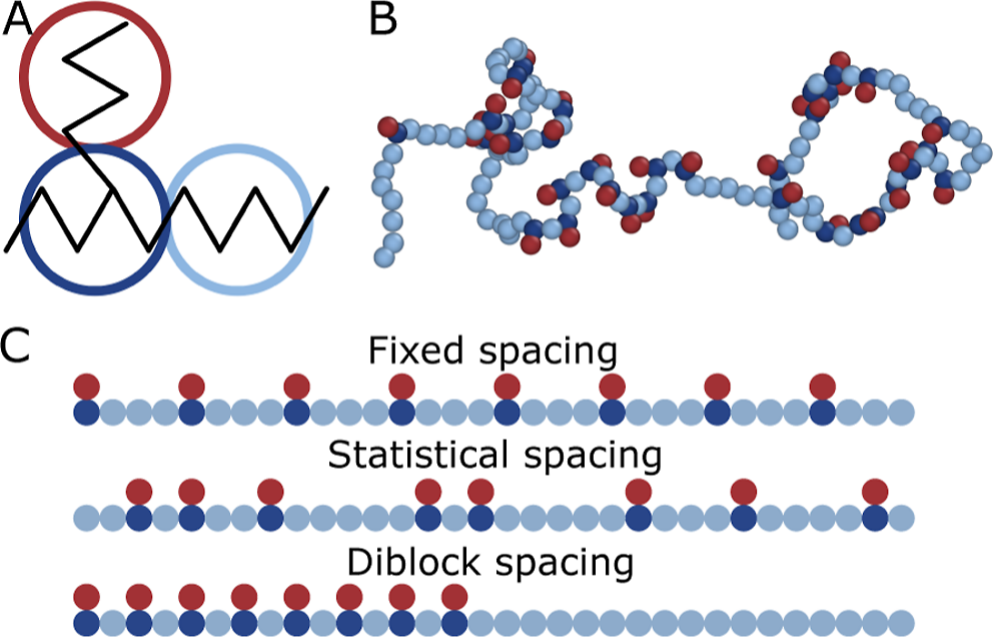
Description of the molecular dynamics model. Illustrates the (A) coarse-grained beads in our model, (B) a snapshot of an LLDPE with statistical branch spacing, and (C) a schematic of the different branch spacing distributions at a branch fraction of *f* = 0.5. Each of the colored coarse-grained beads represent 4 carbons.

In our simulations, all branches have a length of *L* = 4 carbons, which is analogous to the experiments of Thompson and Orski.^28^ We provide a snapshot of one of our simulated LLDPE chains in Figure 1B. We denote the branch fraction (*f*) as the number of monomers with branches divided by the maximum number of branches along the backbone. To keep our simulations consistent with experiments, we maintain a minimum spacing of *S*_min_ = 8 carbons between branches, i.e., *f* = 1 corresponds to every other backbone monomer containing a branch.

We obtain different branch spacing distributions by placing branches along the backbone in various ways. A fixed branch spacing distribution means branches have a fixed spacing of *S* = *S*_min_/*f* carbons apart. Statistical branch spacing indicates that we place branches with some probability (*p*_*B*_) along the polymer’s backbone under the constraints that branch fraction is fixed and no two monomers are less than *S*_min_ apart. A diblock branch spacing distribution means that the first *f* fraction of the backbone monomers are LLDPE with a branch spacing of *S*_min_, and the next (1 – *f*) fraction of backbone monomers are linear polyethylene. We show examples of these branch spacing distributions in Figure 1C for a branch fraction of *f* = 0.5.

We use the large-scale atomic/molecular massively parallel simulator (LAMMPS)^33^ code with a Langevin thermostat and time step of 8 fs. To speed up the rate of exploration of molecular conformations in these simulations, we perform parallel tempering^34^ and average our dilute solution properties over all snapshots and temperatures using the multistate Bennett acceptance ratio estimator.^35^ To ensure that we have a well-mixed sample, we determine the time for the radius of gyration autocorrelation function to decay to e^–1^. We discard observations before the first decorrelation time from our analysis to avoid initialization effects and ensure we simulate all systems long enough so that the radius of gyration decorrelates multiple times to ensure good sampling. We run 10 replicate simulations for each fixed spacing architecture and 25 replicates for our statistical and diblock spacing architecture to obtain statistics. For our statistical LLDPE, we place a new set of branches for each replicate.

We obtain our intrinsic viscosity and the hydrodynamic radius using the ZENO code, where we take the radius of the monomers to be 2.8 Å,^36–39^ commensurate with the size of the coarse-grained beads. This code approximates these values for each snapshot in our simulation using relationships between the hydrodynamic radius and capacitance, as well as intrinsic viscosity and intrinsic conductivity, as detailed in ref 37. This scheme allows the approximation of dynamic quantities like intrinsic viscosity and the hydrodynamic radius without the use of more complex simulation methods seen in other studies.^40^ ZENO exhibits a small systematic error in its computed intrinsic viscosities from experimental values. To address this issue, we multiply all computed intrinsic viscosity values by a constant of *c* = 0.68, as in our previous paper.^26^

## Results and Discussion

### Theoretical Modeling

#### Fixed Branch Spacing

We now summarize our previous theory^26^ for LLDPE with fixed branch spacing. We initially consider our expression for the radius of gyration

3 $$R_{g,f}=\lambda_{R_{g}}K_{R_{g}}^{l}{\left(\gamma M\right)}^{\nu_{R_{g}}}$$

where $K_{R_{g}}^{l}$ is the scaling prefactor of the linear polymer, ν_*R*_g__ is the scaling exponent of the linear polymer, and

4 $$\gamma =1/\left(1+M_{L}/M_{S}\right)$$

rescales the macromolecule mass (*M*) so that it is the same as a linear polymer with the same backbone length. In particular, *M*_L_ is the mass of the branch, and *M*_S_ is the mass of the chain between branches. The final term, λ_*R*_g__, accounts for the changes in the persistence length of the chain due to two sources: branch-backbone (δ_k_) and branch–branch (δ_r_) excluded volume effects. We write this term as λ_*R*_g__=(δ_k_ δ_r_)^2/5^, where the power accounts for the change in Kuhn length from the branches according to Flory theory.^13^

To obtain an analytic form of λ_*R*_g__, we first take into account the changes due to excluded volume interactions between the branches and backbone. These interactions cause the backbone to kink, decreasing the persistence length of the polymer. Using a modification of the freely rotating chain model, we show the ratio of the branched to unbranched persistence length of the polymer due to this effect equals

5 $$\delta_{k}=\frac{\ln\left(\langle \cos\left(\theta_{XAX}\right)\rangle \right)}{\ln\left(4/S\left\langle \cos\left(\theta_{XBX}\right)\right\rangle +\left(1-4/S\right)\left\langle \cos\left(\theta_{XAX}\right)\right\rangle \right)}$$

where ⟨ cos­(θ_XBX_)⟩ and ⟨ cos­(θ_XAX_)⟩ are the average cosine angles along the backbone of the branched and unbranched monomers measured from our molecular dynamics simulations, respectively. Here, S is the spacing between branches, which we measure in carbons for convenience. The factors of 4 come from 4 carbons being in a single monomer in our coarse-grained model.

Next, we consider changes in the persistence length due to repulsive interactions between branches. Previously, we argued that the ratio of persistence length from the branched to the unbranched polymer due to this repulsion is $\delta_{r}=1+C{\left(\frac{L}{S}\right)}^{6/5}$ where *C* is a fitting constant. While this form fit the original data reasonably well, we observed that when we increased the number of replicas, decreasing the noise in our data, an apparent deviation occurred from the new data. Because the exact nature of this term is still debated, and it may be a function of chain stiffness,^41^ we now argue for a different form than was previously suggested, which is more consistent with our data. In particular, a mean-field Flory-type approach suggests δ_r_ ∼*L*/*S* for *L* ≫ *S*.^20^ Because the branch–branch interaction has a finite range, we suggest the form

6 $$\delta_{r}=1+C_{1}\max\left(0,L/S-C_{2}\right)$$

Here, *C*_1_ modulates the strength of the repulsive term, and *C*_2_ dictates the range of the branch–branch interactions, i.e., *L* > *C*_2_ *S* before repulsive interactions occur. We must fit these values to the data; here, we find *C*_1_ = 0.8 and *C*_2_ = 0.3. This value of *C*_2_ matches our intuitive expectation that 0 < *C*_2_ < 1/2, where the 1/2 comes from nearest-neighbor branches needing to be less than *S* = 2*L* to regularly interact. Moreover, it matches the spirit of Tang et al.^42^ and Sunday et al.,^27^ who have recently presented evidence that branch–branch excluded volume interactions do not become appreciable until *L* > 3.5*S* and *L* > *S*, respectively. Moreover, this functional form causes our radius of gyration function to scale as (*L*/*S*)^2/5^, roughly in line with experiments from Pan et al. and theory from Birshtein et al., who found this scaling to be (*L*/*S*)^0.37^ and (*L*/*S*)^9/25^, respectively.^23,24,43^ This form is consistent with our prior fixed spacing data for *L* > 2 carbons as shown in Figure S1 of the Supporting Information.

To determine the other dilute solution properties, we noted empirical power-law relationships, $g_{\left[\eta \right]}=g_{R_{g}^{2}}^{\varepsilon_{\left[\eta \right]}}$ and $g_{R_{h}}=g_{R_{g}^{2}}^{\varepsilon_{R_{h}}}$ , were constant for comb-like polymers with fixed branch spacing and *L*≫̷S. Thus

7 $$\left[\eta \right]=\lambda_{\left[\eta \right]}K_{\left[\eta \right]}^{l}{\left(\gamma M\right)}^{\nu_{\left[\eta \right]}}$$

$$R_{h}=\lambda_{R_{h}}K_{R_{h}}^{l}{\left(\gamma M\right)}^{\nu_{R_{h}}}$$ 8

where λ_[η]_ = γ^–ν_[η]_^ $(\lambda_{R_{g}}\gamma^{\nu_{R_{g}}}{)}^{{2\varepsilon }_{\left[\eta \right]}}$ and $\lambda_{R_{h}}=\gamma^{{-\nu }_{R_{h}}}(\lambda_{R_{g}}\gamma^{\nu_{R_{g}}}{)}^{{2\varepsilon }_{R_{h}}}.$ These equations allow the prediction of fixed branch spacing architecture directly from experimentally measurable dilute solution properties.

In our original paper,^26^ we fit ε_[η]_ and ε_*R*_h__ in these equations using the model that we developed. Because we have modified our previous model’s δ_r_, we refit these parameters to the power-law relationships $g_{\left[\eta \right]}=g_{R_{g}^{2}}^{\varepsilon_{\left[\eta \right]}}$ and $g_{R_{h}}=g_{R_{g}^{2}}^{\varepsilon_{R_{h}}}$ using the original data, as shown in the Supporting Information, Figure S3. This more straightforward approach provides ε_[η]_ = 1.196 ± 0.005 and ε_*R*_h__ = 0.362 ± 0.005, slightly higher than the previously reported ε_[η]_ = 1.13 ± 0.03 and ε_*R*_h__ = 0.33 ± 0.02. The error presented is the standard error. We use these updated contraction factor exponents throughout the rest of the manuscript.

#### Statistical Branch Spacing

In the case of a comb-like polymer in which branches are placed randomly along the backbone, we apply the theory for fixed branch spacing to the distribution of branch spacings along the backbone. Thus, we first must determine the distribution of the number of branches *P*(*N*_B_), where *N*_B_ is the number of branches.

Polymers with statistical branch spacing have main chain monomers along the backbone of mass *M*_A_, branch monomers of mass *M*_B_, and branches of mass *M*_L_. When the total mass is the sum of the branched and the unbranched monomer masses, *M* = (*N* – *N*_B_)*M*_A_ + *N*_B_(*M*_B_ + *M*_L_) where *N* is the backbone length in monomers. If *M*_B_ + *M*_L_ ≠ *M*_A_, we can equivalently state that the backbone length is a function of the number of branches

9 $$N=\frac{M-N_{B}\left(M_{B}+M_{L}-M_{A}\right)}{M_{A}}$$

To determine the distribution of *N*_B_, we note that every monomer along the backbone must have the same probability of branching p_B_. The binomial theorem tells us that the likelihood of having *N*_B_ branches given a backbone of length *N* is

10 $$P\left(N_{B}\right)\underset{∼}{\propto }\left(\begin{array}{l} N\\ N_{B} \end{array}\right)p_{B}^{N_{B}}{\left(1-p_{B}\right)}^{N-N_{B}}$$

Here, the approximate proportionality rather than equality stems from fixing *M* rather than fixing *N*. This constraint disallows some chain conformations to ensure eq 9 holds. As *M* → ∞, this expression simplifies to

11 $$P\left(N_{B}\right)=\frac{M_{A}+f\left(M_{B}-M_{A}+M_{L}\right)}{M_{A}\sqrt{2\pi Nf\left(1-f\right)}}\exp\left(-\frac{{\left(N_{B}-fN\right)}^{2}}{2Nf\left(1-f\right)}\right)$$

Additional information on eqs 10 and 11 is in the Supporting Information.

Having found the distribution of the number of branches, we turn to the distribution of branch spacing at a given number of branches, *P*(*S*|*N*_B_). Imagine that a comb-like polymer has two branches that are *S* monomers apart. There are $\left(\begin{array}{c} N-S-1\\ N_{B}-2 \end{array}\right)$ ways to place the other *N*_B_ – 2 branches along the other *N* – *S* – 1 backbone monomers. We can repeat this argument for all *N* – *S* intervals of length *S* along the backbone. Thus, the number of configurations with branches that are *S* apart is

12 $$P\left(S|N_{B}\right)\propto \left(N-S\right)\left(\begin{array}{c} N-S-1\\ N_{B}-2 \end{array}\right)$$

Now that we have determined the necessary distributions, we compute a comb-like polymer’s dilute solution properties. We use our fixed branch distribution theory to obtain γ and δ_*k*_ (eqs 4 and 5) by substituting the average branch spacing *S* = *S*_min_ *N*/*N*_B_ or *M*_S_ = *M*_S,min_ *N*/*N*_B_, where *S*_min_ and *M*_S,min_ is the minimum distance and chain mass between branches. This substitution is valid because these terms only use *S* to determine the number of branches along the chain, not their position. On the other hand, the strength of the repulsive interactions between branches (δ_r_) depends on how close the nearest neighbor branches are to one another. As such, we take the average of the repulsive interactions between neighboring branches, ⟨δ_r_⟩ = *∑*_S_ *P*(*S*|*N*_B_)­δ_r_(*L*, *S*), to describe the strength of this repulsion for statistical comb-like polymers. In this case, we may describe the radius of gyration for statistical copolymers as

13 $$R_{g,s}=\sum_{N_{B}}P\left(N_{B}\right)\lambda_{R_{g,s}}K_{R_{g}}^{l}{\left(\gamma M\right)}^{\nu_{R_{g}}}$$

where $\lambda_{R_{g,s}}={\left(\delta_{k}\left\langle \delta_{r}\right\rangle \right)}^{2/5}$ .

As the persistence length is relatively constant along the chain’s backbone, we expect these statistical polymers to have scalings similar to the fixed branch spacing distribution polymers. As such, we anticipate that the scaling relationships demonstrated in eqs 7 and 8 can be used to obtain expressions for intrinsic viscosity and the hydrodynamic radius.

#### Diblock Branch Spacing

In the previous spacing distributions, the persistence length of the chain remains relatively constant along the length of the chain. A diblock linear-comb polymer contains two distinct persistence lengths: one for the linear segment and one for the branched segment. Thus, it requires special treatment. In particular, Douglas and Freed^30^ have estimated the self-avoiding limit of the radius of gyration by renormalization group theory and the ϵ expansion as

14 $$R_{g,d}^{2}=xR_{g,f,c}^{2}+\left(1-x\right)R_{g,f,l}^{2}+\xi \left(R_{g,f,c}^{2}+R_{g,f,l}^{2}\right)$$

where

15 $$\xi =\frac{1-x^{2\nu_{R_{g}}+1}-{\left(1-x\right)}^{2\nu_{R_{g}}+1}}{x^{2\nu_{R_{g}}}+{\left(1-x\right)}^{2\nu_{R_{g}}}}$$

*x* = *M*_c_/*M* = *f*/(*f* + (1 – *f*)*M*_A_/(*M*_L_ + *M*_B_)) is the mass fraction of the comb-like block, *R*_g,f,c_^2^ is the radius of gyration of an comb-like block with fixed branch spacing and mass *M*_c_, and *R*_g,f,l_^2^ is the radius of gyration of the linear block with mass *M*_*l*_ = *M* – *M*_c_. Given the two distinct persistence lengths of these diblocks, the empirical scaling laws for [η] and *R*_h_ as in eqs 7 and 8 used in previous sections no longer hold. Thus, alternative approaches that are beyond the scope of this manuscript are required.^29,44^

### Contraction Factors for Different Comb-like Polymer Branch Spacing Distributions

We now compare Mark–Houwink plots determined through molecular dynamics simulations to prior experimental work in Figure 2A,B. Specifically, Orski et al.^17^ measured the intrinsic viscosity of linear polyethylene (red) and an LLDPE (dark blue) with fixed branch length and spacing of *L* = 4 and *S* = 8 carbons. These lines appear in Figure 2A,B as they represent branch fractions of *f* = 0 and *f* = 1, which occur in both cases. Additionally, we plot diblock and statistical branch spacing intrinsic viscosity data with branch lengths of *L* = 4 carbons from Thompson and Orski.^28^ The diblock branch placement has branch fractions of *f* = 0.20 (orange), 0.47 (gray), and 0.75 (light blue), while the statistical branch placement has branch fractions of *f* = 0.20 (orange), 0.52 (gray), and 0.67 (light blue). We perform corresponding molecular dynamics simulations at the same branch length and similar branch fractions of *f* = 0, 0.25, 0.50, 0.75, and 1 from red to blue. These simulations are represented as points at low molecular mass.

**Figure 2:**
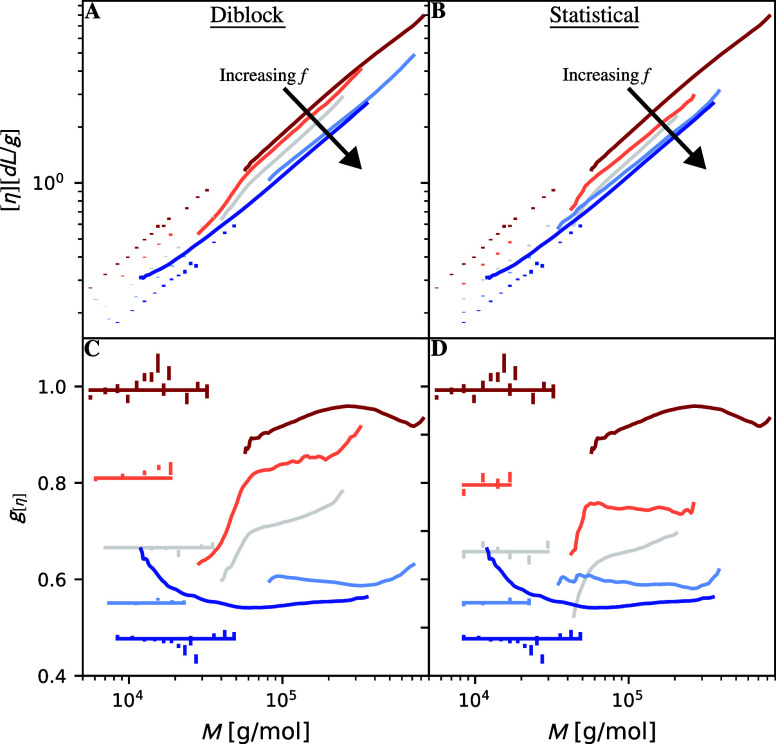
Comparison between experimental and simulation contraction factors. (A,B) compare Mark–Houwink plots for diblock and statistical LLDPE with branch lengths of four carbons. The points at low *M* come from molecular dynamics simulations, while the lines at high *M* are taken from experiments.^17,28^ Colors from red to blue represent branch fractions of approximately *f* = 0, 0.25, 0.5, 0.75, and 1.0. The red and blue data for *f* = 0 and *f* = 1 correspond to the cases in which the polymers have no branches and the maximum amount of branches. Thus, these sets of data are the same in panels (A,B). (C,D) show the intrinsic viscosity contraction factors of the data from (A,B) normalized by the polyethylene scaling law of the molecular dynamics simulations. The horizontal lines through the simulated data represent the average contraction factor across all simulated data. Error bars on the simulated data represent bootstrap error in the mean of all independent molecular dynamics replicates.

Figure 2A,B show that the experimental and molecular dynamics intrinsic viscosities scale similarly, i.e., they have the same scaling exponent, regardless of branch spacing distribution or spacing. To highlight changes in the scaling behavior of Figure 2A,B, we now consider their contraction factors (*g*_[η]_) in Figure 2C,D. For consistency, we normalize all relationships by the power-law relationship to fit the intrinsic viscosity of the molecular dynamics simulations for linear polyethylene with its apparent exponent set to ν_[η]_ = 0.684 ± 0.005, as measured in a previous publication^26^ and in line with expectations.^13^ Here, our simulated data lies at a constant contraction factor for each case, while the experimental data shows substantial deviations from these values at low and high molecular mass. These deviations are likely due to measurement error and long-chain branching in the synthesis.^17,28^ Even under this strict comparison between experimental and simulated data, the simulations obtain nearly quantitative agreement with the experiments when we use our previously computed correction constant *c*. These results bolster our previous work,^26^ which showed that our molecular dynamics model can reproduce the intrinsic viscosity scaling of LLDPE with fixed branch spacing.

We also note that *g*_[η]_ is nearly constant with *M*. This observation also holds for the contraction factors of the radius of gyration and hydration, as shown in the Supporting Information Figure S2. It suggests that all the comb-like polymers we study reside in the same scaling regime, i.e., the scaling exponent of each property is constant as a function of branch fraction and branch spacing distribution. This result is analogous to our previous finding that scaling exponents of these polymers are the same for fixed branch spacing and length^26^ and lies in contrast to when *L* ≫ *S* (the bottlebrush regime). In the bottlebrush regime, we expect the polymer transforms from a linear coil to a rod as *S* → 0 increasing the scaling exponents. As these contraction factors are constant, we can take the average of these contraction factors across all masses as the contraction factor for a given architecture.

To determine trends in the contraction factor data, we plot branch fraction (*f*) against the average contraction factor for all our dilute solution properties in Figure 3. Because the means of the contraction factors are plotted in Figure 2C,D, the error in the data is smaller than the point size. In all cases, as branch fraction increases, contraction factors decrease. Interestingly, fixed branch spacing leads to smaller contraction factors at all studied branch fractions. Regarding diblock and statistical branch spacing, these properties show substantial differences in their radius of gyration contraction factors, in which the diblock data exhibits a clear S-shaped curve in Figure 3A. On the other hand, the differences between these data sets are much more subtle for intrinsic viscosity and the hydrodynamic radius. While the slight increase in intrinsic viscosity when comparing statistical and diblock branch spacing at a branch fraction of *f* = 0.25 in Figure 3B is in qualitative agreement with experiments, the experiments did show a somewhat stronger effect. We are unsure whether these differences are due to experimental noise or issues obtaining intrinsic viscosity using ZENO. To better understand this data, we use the theoretical models as detailed in prior section. We plot these lines in Figure 3. These lines capture the overall trends in the dilute solution properties well. Having established the correspondence between theory and simulation, we can now use theory to explain the trends in the simulated data. As *f* increases, the chain’s backbone length decreases while the number of kinks increases, leading to the mass rescaling (γ) and kink (δ_k_) factors’ decrease. These decreases are the primary factors in the reduction of all dilute solution properties with *f*.

**Figure 3:**
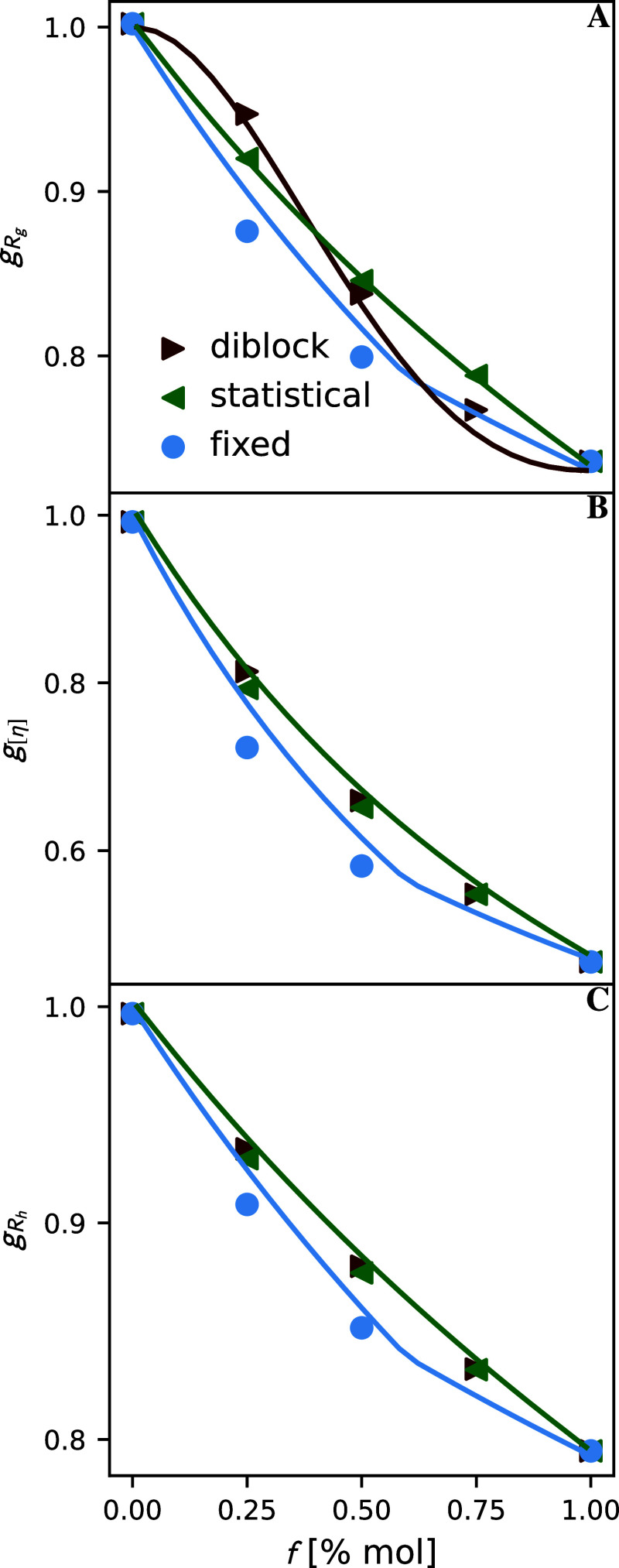
Mean contraction factors for differing branch distributions and branch fractions. Shows contraction factors of (A) the radius of gyration, (B) intrinsic viscosity, (C) the hydrodynamic radius as a function of the fraction of branching monomers. The red rightward-facing triangles, green leftward-facing triangles, and circles represent diblock, statistical, and fixed spacing branch distributions. Points represent molecular dynamics simulations. Lines represent the theory explained in the Theoretical modeling section. The error in the data is smaller than the point size.

To explain the difference between the fixed and statistical spacings, we note that γ is fixed at a given *f*. Moreover, the standard deviation of the distribution of the number of branches *P*(*N*_B_) (eq 11) grows sublinearly with backbone length (*N*), yielding only minor changes in *N*_B_/*N* as polymer mass *M* → *∞*. Therefore, for large *M*, δ_*k*_ remains approximately constant, and we expect the most considerable changes to derive from the larger repulsion factor (δ_r_) for the statistical copolymer due to the presence of more close branches along the backbone.

We now move to the S-shaped curve in the *g*_*R*_g__ plot for the diblock PE-LLDPE. The diblock *R*_g_ theory proposed by Douglas and Freed (eq 14) suggests that $\frac{dR_{g,d}^{2}}{df}\left(x=0\right)=\frac{dR_{g,d}^{2}}{df}\left(x=1\right)=0$ for any diblock in good solvent at mass fraction of the comb-like block *x*. This behavior necessitates the S-shaped curve in the diblock *g*_*R*_g__ and the intersection of this curve with the fixed and statistical lines. To understand this equation physically, we can exactly rewrite the diblock radius of gyration as

16 $$R_{g,d}^{2}=xR_{g,f,c}^{2}+\left(1-x\right)R_{g,f,l}^{2}+x\left(1-x\right)\left\langle G^{2}\right\rangle$$

where, as mentioned previously, *R*_g,f,c_^2^ and *R*_g,f,l_^2^ are the radius of gyration of the comb-like and linear blocks of the diblock and ⟨*G* ^2^⟩ is the mean squared distance between the centers of mass of the blocks.^31^ This is illustrated in Figure 4. By taking the derivative of this expression with respect to *x* at *x* = 0 (a linear chain) and setting it to 0, we obtain

17 $$\left\langle G^{2}\right\rangle \left(x=0\right)=\left(2\nu_{R_{g}}+1\right)R_{g,f,l}^{2}\left(x=0\right)$$

In this case, we can interpret ⟨*G* ^2^⟩(*x* = 0) as the mean squared distance between the center of mass and the chain end. As anticipated, this equation shows that ⟨*G* ^2^⟩(*x* = 0) scales with the radius of gyration of the dominant block, i.e., ⟨*G* ^2^⟩(*x* = 0) ∼ *R*_g,f,l_^2^(*x* = 0), the only relevant length scale of the problem. The constant (2ν_*R*_g__+ 1) indicates ⟨*G* ^2^⟩(*x* = 0) > *R*_g,f,l_^2^(*x* = 0), as expected for a segments at the chain ends rather than chain center. This intuitive behavior is consistent with the theory of Douglas and Freed and similar behavior can be shown for *x* = 1.

**Figure 4:**
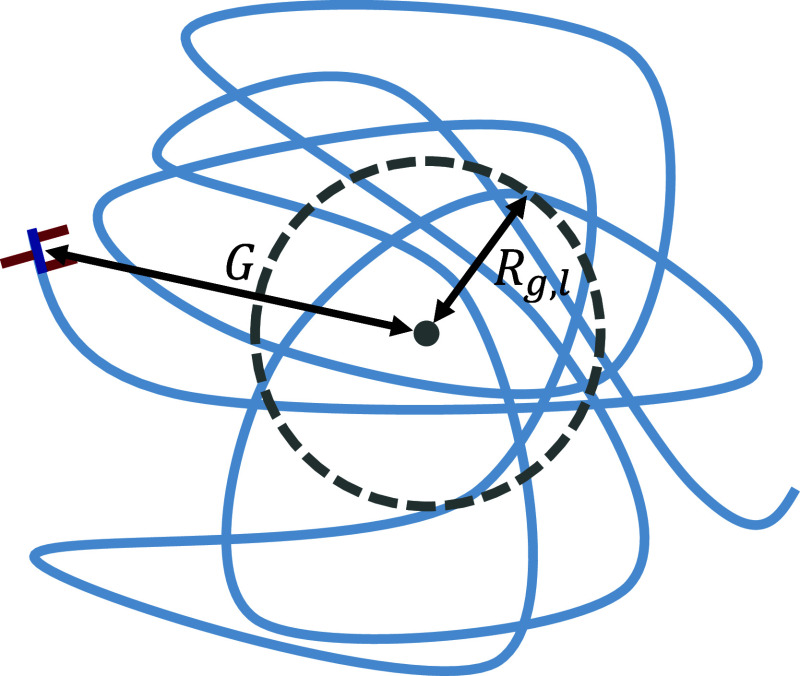
Schematic of diblock polymer near *x*= 0. Illustrates a majority linear (light blue) diblock polymer with a small LLDPE with precise spacing (dark blue with red branches) at the end. Here, *R*_g,l_ is the radius of gyration of the linear block from the center of mass of the linear segment (the gray dot) and *G* is the distance between the linear and LLDPE centers of mass.

### Relationship between Dilute Solution Properties and the Freely Rotating Chain Persistence Length

While we have shown that our model can predict chain-level dilute solution properties, we have yet to show how these relationships relate to the chain’s microscopic properties. We consider the dilute solution properties of fixed and statistical branch distribution polymers via eqs 3–8 and 13 to probe these local chain properties. We do not consider the diblock branch spacing in this section due to the presence of two persistence lengths in the chain, one for each block. By rearranging these equations, we find that the contraction factors of the fixed and statistical branch spacing distributions are related to the macromolecule’s persistence length (*l*_p_) in the following ways

18 $$\lambda_{R_{g}}\left(l_{p}\right)=g_{R_{g}}\gamma^{-\nu_{R_{g}}}$$

19 $$\lambda_{R_{g}}\left(l_{p}\right)=g_{\left[\eta \right]}^{1/\left(2\varepsilon_{\left[\eta \right]}\right)}\gamma^{-\nu_{R_{g}}}$$

20 $$\lambda_{R_{g}}\left(l_{p}\right)=g_{R_{h}}^{1/\left(2\varepsilon_{R_{h}}\right)}\gamma^{-2\nu_{R_{g}}}$$

where λ_*R*_g__(*l*_p_) is only a function of *l*_p_. Thus, these equations represent different methods of measuring the same function of persistence length from the contraction factors of the radius of gyration, intrinsic viscosity, and hydrodynamic radius, respectively.

Intuitively, λ_*R*_g__(*l*_p_) represents the contraction factor of the radius of gyration for branched and linear polymers of the same backbone length, rather than the same mass. Thus, λ_*R*_g__(*l*_p_) < 1 indicates kinking from branches interacting with the backbone causes the branched polymer *R*_g_ to contract compared with a linear polymer with the same backbone length. On the other hand, λ_*R*_g__(*l*_p_) > 1 indicates interactions between branches causes the branched polymer to grow compared to a linear polymer.

While we have implicitly postulated this relationship, we have yet to confirm this relationship directly. To do so, we compute the persistence length of the freely rotating chain model

21 $$l_{p}^{FJC}=l_{b}s$$

where *l*_b_ is the bond length and *s* is the chemical distance such that

22 $$\left\langle \vec{b_{i}}\cdot \vec{b_{i+s}\;}\right\rangle /l_{b}^{2}=e^{-1}$$

Here, $\vec{b_{i}}$ is the bond vector of *i* and e is base of the natural logarithm. Recent work has found that $\left\langle \vec{b_{i}}\cdot \vec{b_{i+s}\;}\right\rangle$ decays as a power law for dense melts,^45,46^ at the Θ point,^47^ and in good solvents,^48^ suggesting that no intrinsic decay length exists for this quantity. These results make the definition of *l*_p_^FJC^ somewhat arbitrary. While taking persistence length to be proportional to ⟨*R*_*g*_^2^⟩ is more consistent,^49^ this definition describes the macrostate of the chain. Thus, it loses the local character of *l*_p_^FJC^, defined in terms of the decorrelation of local bonds. Thus, despite its deficiencies, we will consider *l*_p_^FJC^ throughout the rest of this section to probe the microscopic state of the polymers.

Figure 5 plots eqs 18–20 against *l*_p_^FJC^ normalized by the linear freely jointed chain persistence length (*l*_*p*,l_^FJC^). This plot shows reasonable scaling collapse across architectures (fixed and statistical branch spacing) and branch fractions (*f*). We anticipate this collapse across the dilute solution properties (varying colors) because eqs 18–20 all measure the same property. We expect this collapse for varying distributions (different shapes) due to the nearly constant persistence length along the backbone. This correspondence bolsters our mathematical arguments. Although our results apply to the comb-like regime, they align with prior simulations in the bottlebrush regime that have shown persistence lengths increase with increased branch length, which is correlated with increased radius of gyration,^25,49^ lending credence to both sets of studies. The significance of our work is twofold. First, it demonstrates the one-to-one relationship between persistence length and the radius of gyration, which does not break when switching from a fixed, e.g., regular/comb and block, to a statistical distribution. Second, it shows that other properties, namely the intrinsic viscosity and hydrodynamic radius, exhibit the same one-to-one correspondence.

**Figure 5:**
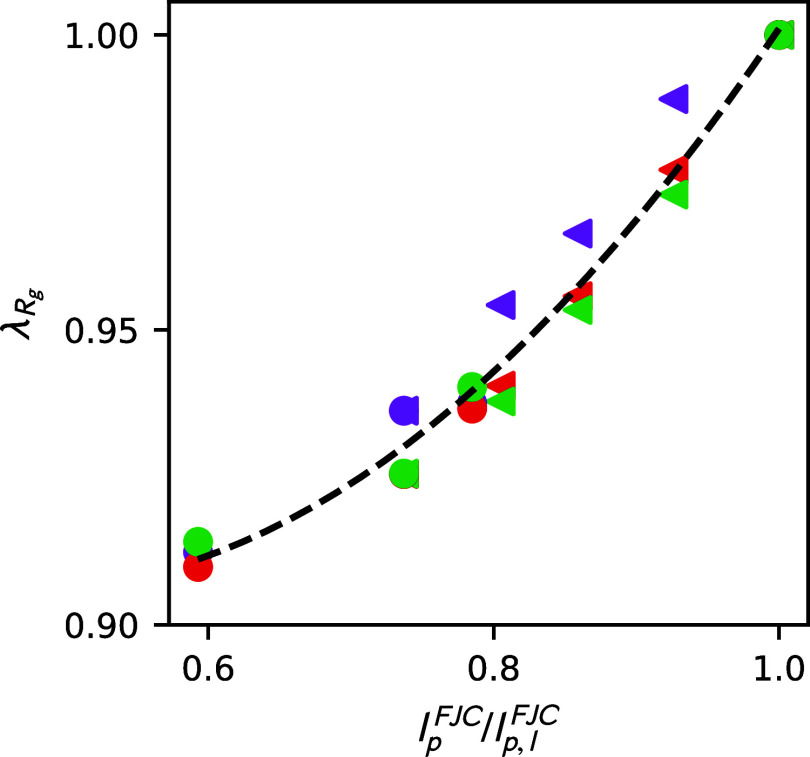
Relationship between contraction factors and freely jointed chain persistence length. Shows the one-to-one relationship between the freely jointed chain persistence length and the contraction factors of the radius of gyration (purple), intrinsic viscosity (red), and the hydrodynamic radius (green), as predicted by eqs 18–20. Circles and leftward-facing triangles denote fixed and statistical branch spacing, respectively. The dashed line provides a guide to the eye. The error in the data is smaller than the point size.

Interestingly, this plot suggests a method to determine the persistence length of a comb-like macromolecule from its macroscale dilute solution properties. Experiments can readily access all properties measured in eqs 18–20. As such, eqs 18–20 provide a method to interrogate the rate of bond decorrelation directly from experimental data.

## Conclusions

This work investigates how differences in branch spacing distributions change the dilute solution properties of comb-like polymers. Using a previously developed molecular dynamics force-field that replicates LLDPE in a good solvent,^26^ we determined the dilute solution properties of these polymers with fixed, statistical, and diblock branch spacing distributions at various branch fractions (side chain densities) ranging from *f* = 0 (linear polyethylene) to *f* = 1 (a precise LLDPE with a branch spacing of *S* = 8 carbons). We compared these simulations against similar experiments by Thompson and Orski.^28^ We found them to match reasonably well, bolstering our confidence in the ability of this force field to replicate experimental data. We then used this data to reaffirm previously developed theories for fixed and diblock branch spacing comb-like polymers in dilute solutions. Next, we extended our previously developed framework to explain the dilute solution properties of polymers with a statistical distribution of branch spacings.^26^ We demonstrated that it describes the simulated data. Finally, we showed that in the cases of fixed and statistical branch spacings, we can relate the persistence length of the polymers directly to the contraction factors of their properties.

These findings offer valuable insights into the behavior of comb-like polymers in dilute solution, paving the way for improved structure–property relationships and a deeper understanding of chain architecture. Additionally, they suggest exercising caution when evaluating the scaling of dilute solution properties, such as the radius of gyration, as a function of only the average branch length and spacing while ignoring the distribution of branch spacing. Although our results are for the comb-like regime, they hint that the distribution of branch spacing will also affect the dilute solution properties of bottlebrush polymers (*L* ≫⟨*S*⟩).

Still, several questions linger. We find an unusually small difference in simulated intrinsic viscosity and hydrodynamic radius for diblock and statistical branch spacing distributions in contrast with earlier experimental work from Thompson and Orski.^28^ These results indicate that diblocks have a moderately higher *g*_[η]_ at *f* = 0.25 and 0.5. We cannot determine whether this difference results from the approximation we are using to measure intrinsic viscosity, ZENO, or this difference has to do with experimental error. We also needed to update our previous theory’s repulsive term (δ_r_) scaling to match our simulated data well.^26^ While we have noted that the new form is quite similar to others, this change highlights the need for high-quality studies to determine this scaling.^24^

Beyond these slight inconsistencies, this study primarily considered three branch spacing distributions at a fixed, relatively short branch length (*L* = 4 carbons). While this work represents an initial step in understanding how varying the distribution of branches affects dilute solution properties, further work is required to test these findings for longer branches, particularly as branch length transitions from short alkyl chains to long-chain branches, i.e., as the mass averaged molecular mass approaches entanglement molecular mass (*M*_w_ → *M*_e_), and to understand other branch spacing distributions, e.g., gradient branch distributions. Moreover, although having a fixed branch length in comb-like polymers is common, this is not always the case. As such, one could also consider distributions of branch lengths in future models.

## Supplementary Material



## Notes

Additional information on theoretical consistency of the new repulsion term with previous data, a derivation of the probability of having *N*_*B*_ branches at a fixed mass *M*, the contraction factors of [η] and *R*_*h*_ as functions of mass, and refitting the contraction factor relationships. Data: study data and coarse-grained force field parameters are available at 10.18434/mds2-2494 (PDF)
