# Supplementary material for: The Importance of Branch Placement on the Dilute Solution Properties of Comb-like Macromolecules
Source: Macromolecules. 2025 Jun 12;58(12):6124–33. doi: 10.1021/acs.macromol.5c00323 (PMC12199475; doi:10.1021/acs.macromol.5c00323)
Supplement: Supplementary file 1 [file ma5c00323_si_001.pdf]

Supporting Information

The Importance of Branch Placement on the Dilute Solution Properties of Comb-like Macromolecules

Robert J. S. Ivancic[1,*], Chase B. Thompson[1], Devin A. Golla[2], Bintou Koroma[3], Jack F. Douglas[1], Sara V. Orski[1], and Debra J. Audus[1]

[1]Materials Science and Engineering Division, National Institute of Standards and Technology, Gaithersburg, Maryland 20899, United States
[2]Department of Chemical and Biomolecular Engineering, University of Pennsylvania, Philadelphia, Pennsylvania 19104, United States
[3]Fischell Department of Bioengineering, University of Maryland, College Park, Maryland 20742, United States
[*]Corresponding author: robert.ivancic@nist.gov

1 Consistency of new repulsion term with prior data

While the form of δ_r proposed in Eq. 6 of the Main Text works well for our most recently collected data, this data set is relatively limited. In particular, we limited ourselves to branches of length $L = 4$ carbons with only a few branch fractions. In our previous publication[26], we simulated linear low-density polyethylene (LLDPE) with fixed branch spacings ranging from $S = 8$ to 32 carbons and branch lengths from $L = 2$ to 12 carbons. This dataset provides a much more robust test of the new repulsive term, especially because it contains many simulations with $2L > S$. As such, we plot the measured dilute solution properties from these simulations against the properties predicted with the modified δ_r in Fig. S1. For Figs. S1B and S1C, we use the contraction factor exponents $\epsilon_{[\eta]}$ and ϵ_{R_h} found in Sec. S4 below. Most data (blue) shows excellent correspondence between the model and the measured values. When $L = 2$ carbons (red), a slight systematic deviation on the order of 10% occurs. While it is unclear why this bias occurs, one possibility is that minor differences between the 2 carbon branching monomers (C_2) from the 4 carbon branching monomers (C_4, depicted in Fig. 1A of the Main Text) cause subtle changes in conformation for short branches.

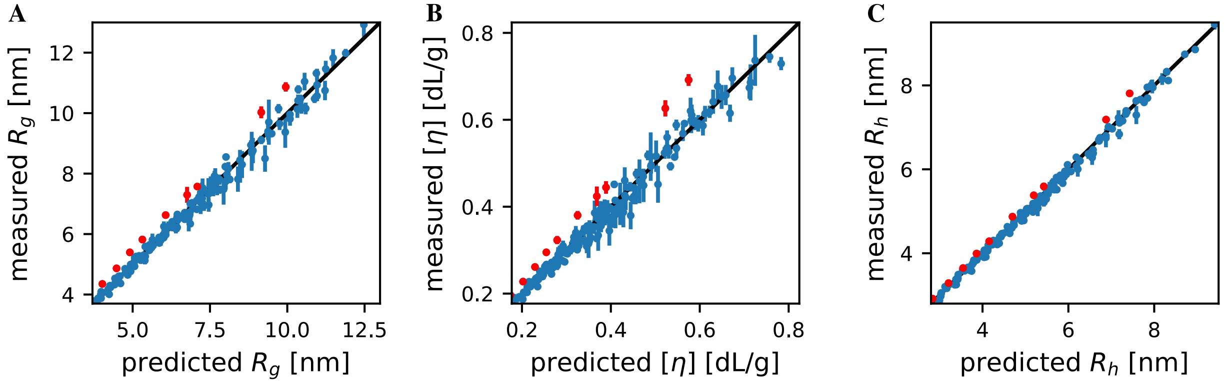

Figure S1: **Measured against predicted dilute solution properties with modified δ_r.** Shows the measured and predicted (**A**) R_g, (**B**) $[\eta]$, and (**C**) R_h with updated δ_r. Blue points are include most data, while red points are data for $L = 2$ carbons case. Error bars are one standard error.

2 Probability of having N_B branches given a polymer mass M (Eqs. 10 and 11)

It is well known that the number of branches (N_B) at *fixed backbone length* (N) is binomially distributed, *i.e.*, $P(N_B|N) = \binom{N}{N_B} p_B^{N_B}(1 - p_B)^{N-N_B}$ and in the large N case assuming $0 < p_B < 1$, this probability becomes Gaussian, *i.e.* $P(N_B|N) \sim \frac{\exp\left(-\frac{(N_B - p_B N)^2}{2Np_B(1-p_B)}\right)}{\sqrt{2\pi N p_B(1-p_B)}}$. Here, we show that similar expressions hold for *fixed polymer mass* (M) as opposed to fixed N. These expressions are nontrivial because for a comb-like polymer at a given mass, we require $M = M_B N_B + M_A(N - N_B)$, where M_B is the branch monomer mass and M_A is the non-branch monomer mass. Thus, for fixed M, N is a function of N_B if $M_B \neq M_A$.

For simplicity, we assume that M_A, M_B, and M are positive integers. If not, we can take an arbitrarily precise rational approximation of M_A and M_B, find their greatest common denominator, and work in units of this greatest common denominator, which will lead to M_A, M_B, and M being positive integers. Moreover, we assume $M_A \neq M_B$. If it does, the proof is the same as the fixed N case. Finally, we assume $0 < p_B < 1$. If not, the chains are homopolymers.

To derive the probability of having N_B branches given a polymer mass M (Eq. 10), imagine growing a comb-like polymer to a mass of M by adding monomers along the backbone. A monomer that includes a branch occurs with probability p_B while a monomer without a branch occurs with probability $(1 - p_B)$. We can add monomers without restriction until a monomer is reached, at which point the number of A and

B monomers added to the chain is dictated by the mass constraint. Mathematically, this condition occurs when $M - M_g < l$, where $M - M_g$ is the difference between the target mass and the mass of the currently grown chain and $l = \mathrm{lcm}\,(M_A, M_B)$ is the least common multiple of M_A and M_B.

Now, let $P_k(M)$ be the probability of adding k mass to the grown chain to reach the target mass (M), *i.e.*, $P_k(M)$ is the distribution of stopping masses in the restricted regime, $k = M - M_g < l$. Because the chain must end in some way, $\sum_{k=0}^{l-1} P_k\,(M) = 1$. Importantly, we claim $P_k(M)$ does not depend on M as $M \to \infty$. To sketch this assertion, we generalize the problem to allow M to be any positive integer. This can be done because the original problem in which $M = M_B N_B + M_A(N - N_B)$ is a subset of allowing M to be any positive integer, *i.e.*, where $k < l$, but there is not necessarily a way to add integer numbers of M_A and M_B to equal k. In this general case, we can write a linear relationship between $P_k(M)$ and $P_k(M-1)$. This equation comes by noting that there are two ways to achieve a final value of k for M. Either $k-1$ is reached at $M-1$, or $k = l-1$ is reached at $M-1$ and one must increase mass by M_A or M_B to reach the stopping condition for M. Thus, $P_k(M) = (1-\delta_{k,0})P_{k-1}(M-1) + \delta_{k,l-M_A}(1-p_B)P_{l-1}(M-1) + \delta_{k,l-M_B}p_B P_{l-1}(M-1)$. Therefore, there is a transfer (transition) matrix, T, that takes $P_k(M) = \sum_{k'} T_{k,k'}P_{k'}(M-1) = \sum_{k'} \left(T^{M-l}\right)_{k,k'} P_{k'}\,(l)$, where $P_{k'}(l) = \delta_{k',l-M_A}(1-p_B) + \delta_{k',l-M_B}p_B$. For large enough M, this equation will converge to the eigenvector that corresponds to the largest eigenvalue of T. Due to conservation of probabilities, that eigenvalue is 1, proving our claim that the $P_k(M)$ is independent of M as $M \to \infty$. Physically, this proof indicates that the distribution of stopping masses should only depend on where one is in the stopping regime or equivalently, $M - M_g$, instead of M for large enough M.

Now, we can write the probability of having N_B branches at a given mass M as

$$P(N_B|M) = \sum_{k=0}^{l-1} P_k(M) P_b(N_B - \tilde{N}_B(k)|N - \tilde{N}(k)), \tag{1}$$

where $P_b(N_B|N)$ is the probability of growing a comb-like polymer with N_B branches given that it has a backbone length of N. $\tilde{N}_B(k)$ and $\tilde{N}(k)$ are the number of B monomers and total number of monomers that must be added to satisfy M for a given k in the constrained portion ($k < l$) of the chain. Since $P_b(N_B - \tilde{N}_B(k)|N - \tilde{N}(k))$ is in the portion of the chain that is unconstrained, that distribution follows the standard binomial distribution. Because $M \to \infty \implies N \to \infty$, we have via the binomial approximation

$$P_b(N_B - \tilde{N}_B(k)|N - \tilde{N}(k)) \sim \frac{1}{\sqrt{2\pi\left(N - \tilde{N}(k)\right)p_B(1-p_B)}} \exp\left(-\frac{\left((N_B - \tilde{N}_B(k)) - p_B\left(N - \tilde{N}(k)\right)\right)^2}{2\left(N - \tilde{N}(k)\right)p_B\,(1-p_B)}\right). \tag{2}$$

By considering the asymptomatic behavior of this function with N (large M), we find

$$P_b(N_B - \tilde{N}_B(k)|N - \tilde{N}(k)) \sim \exp\left(-\frac{2\tilde{N}_B(k) - \tilde{N}(k)p_B}{2 - 2p_B}\right) \frac{1}{\sqrt{2\pi N p_B(1-p_B)}} \exp\left(-\frac{(N_B - p_B N)^2}{2N p_B\,(1-p_B)}\right) \tag{3}$$

Returning to Eq. 1, we now find

$$P(N_B|M) \sim \frac{1}{\sqrt{2\pi N p_B(1-p_B)}} \exp\left(-\frac{(N_B - p_B N)^2}{2N p_B\,(1-p_B)}\right) \sum_{k=0}^{l-1} P_k(M) \exp\left(-\frac{2\tilde{N}_B(k) - \tilde{N}(k)p_B}{2 - 2p_B}\right) \tag{4}$$

and noting the invariance of $P_k(M)$ as $M \to \infty$, we obtain

$$P(N_B|M) \underset{\sim}{\propto} \frac{1}{\sqrt{2\pi N p_B(1-p_B)}} \exp\left(-\frac{(N_B - p_B N)^2}{2N p_B\,(1-p_B)}\right). \tag{5}$$

Physically, we can think of this as $P(N_B|M)$ being unaffected by the restricted section of growing the polymer chain, since this restricted region would only account for a small amount of the chain as M goes to infinity. Although this expression has the form of the Gaussian approximation of the binomial distribution, it is not. Because M is fixed, N is a function of N_B via Eq. 9 in the Main Text. Nevertheless, using the Stirling

approximation and definitions of binomial coefficients we can obtain Eq. 10 in the Main Text in the exact same manner that we would show the binomial to Gaussian approximation. Thus,

$$P(N_B|M) \underset{\sim}{\propto} \binom{N}{N_B} p_B^{N_B} (1 - p_B)^{N-N_B}, \tag{6}$$

which is equivalent to Eq. 11 in the main text. To obtain the simplified version (Eq. 11 in the main text), we first fix the prefactor so that the integral over all N_B (from $N_B = 0$ to $N_B = M/M_B$) is unity. This yields

$$P(N_B|M) = \frac{M_A + p_B(M_B - M_A)}{M_A\sqrt{2\pi N p_B(1 - p_B)}} \exp\left(-\frac{(N_B - p_B N)^2}{2N p_B(1 - p_B)}\right). \tag{7}$$

Finally, we note that

$$f = \sum_{N_B} N_B P(N_B)/N \approx \sum_{N_B} p_B N P(N_B)/N = p_B. \tag{8}$$

Substituting this relationship into Eq. 5 gives Eq. 11 in the Main Text,

$$P(N_B) = \frac{M_A + f(M_B - M_A)}{M_A\sqrt{2\pi N f(1 - f)}} \exp\left(-\frac{(N_B - fN)^2}{2Nf(1 - f)}\right). \tag{9}$$

3 Contraction factors of other dilute solution properties

We show the contraction factors for the radius of gyration and hydrodynamic radius against molecular mass for diblock and statistical spacing of LLDPE in Fig. S2. These plots show nearly constant contraction factors across all molecular weights, where the linear polyethylene has a value of approximately unity. This plot justifies our use of contraction factors averaged across molecular weight.

4 Refitting contraction factor relationships

Here, we fit our contraction factor exponents $\epsilon_{[\eta]}$ and ϵ_{R_h} in the equations $g_p = g_{R_g^2}^{\epsilon_p}$, where $p = [\eta]$ or R_h. In Fig. S3, we plot contraction factor data from our previous publication[26] with fixed branch spacings. The lines plotted are fits with the exponents $\epsilon_{[\eta]} = 1.196 \pm 0.005$ and $\epsilon_{R_h} = 0.362 \pm 0.005$. The fits show good agreement for the modified exponents.

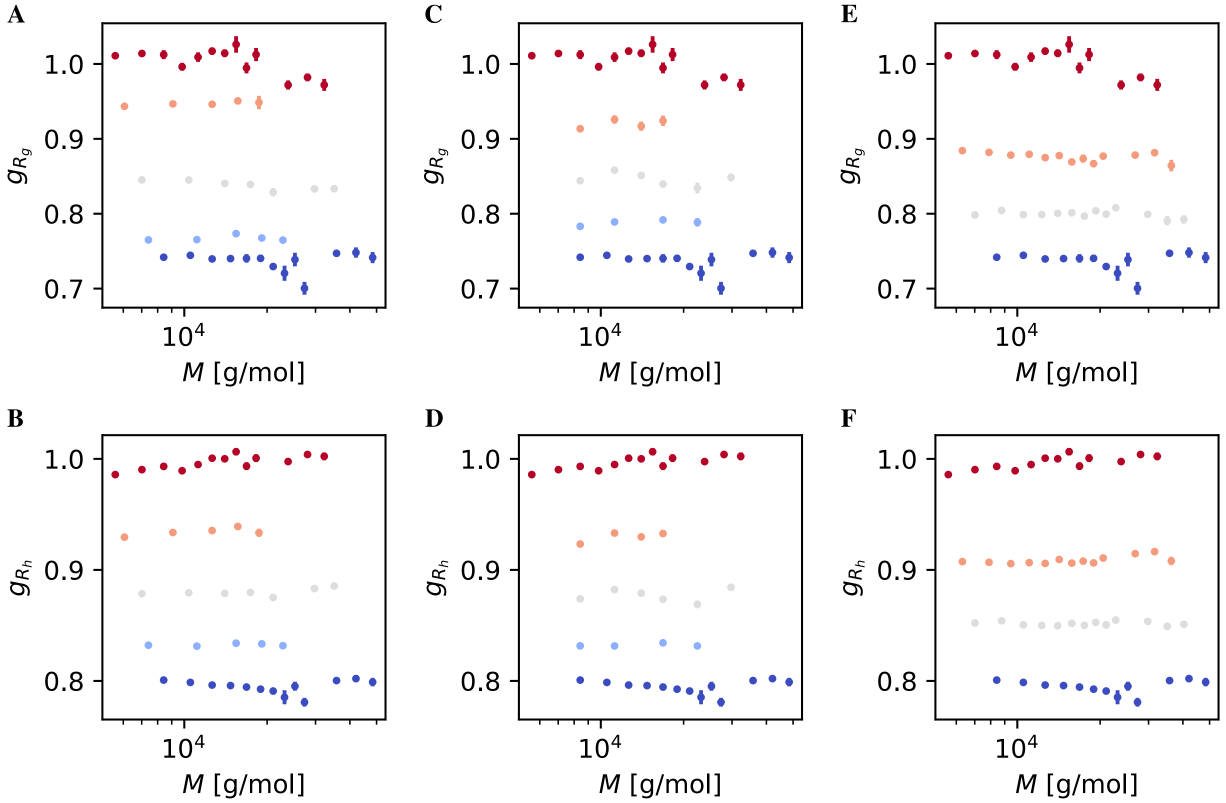

Figure S2: **Contraction factors of R_g and R_h.** Displays the contraction factors of R_g and R_h for the the diblock ((**A**) and (**B**)), statistical ((**C**) and (**D**)), and fixed spacing ((**E**) and (**F**)).

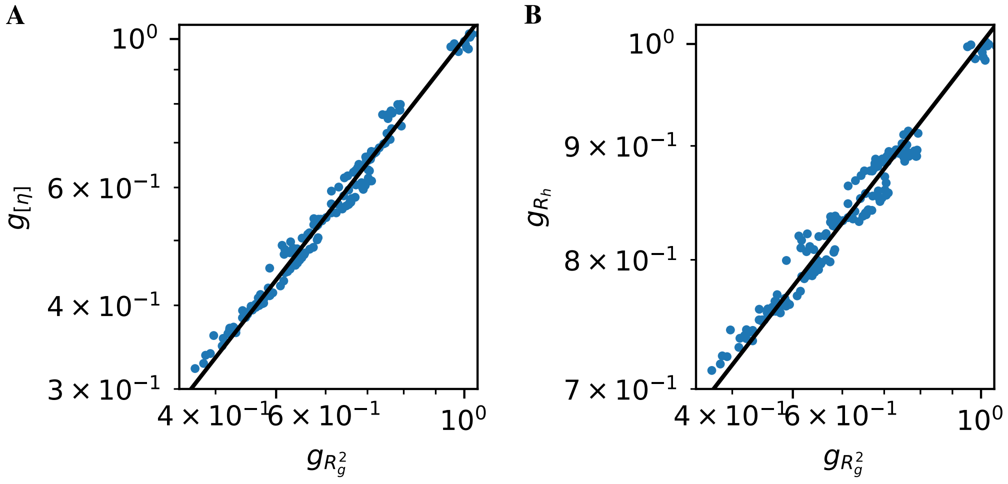

Figure S3: **Relationship between contraction factors.** Shows the power-law relationship between $g_{R_g^2}$ and (**A**) $g_{[\eta]}$ as well as (**B**) g_{R_h}.
